# Supplementary material for: Morphological evolution of language-relevant brain areas
Source: PLoS Biol. 2023 Sep 1;21(9):e3002266. doi: 10.1371/journal.pbio.3002266 (PMC10501646; doi:10.1371/journal.pbio.3002266)
Supplement: S1 Table — (DOCX) [file pbio.3002266.s001.docx]

| **Specimen** | **Sex** | **Age (y)** | **Rearing** |
| --- | --- | --- | --- |
| C0273 | m | 40,0 | Wild |
| C0336 | f | 44,0 | Wild |
| C0342 | f | 35,4 | Wild |
| C0367 | m | 41,2 | Wild |
| C0408 | f | 44,5 | Wild |
| C0301 | m | 35,9 | Captive - Nursery |
| C0423 | m | 24,0 | Captive - Nursery |
| C0491 | m | 18,5 | Captive - Mother |
| C0630 | f | 12,0 | Captive - Mother |
