# Supplementary material for: Morphological evolution of language-relevant brain areas
Source: PLoS Biol. 2023 Sep 1;21(9):e3002266. doi: 10.1371/journal.pbio.3002266 (PMC10501646; doi:10.1371/journal.pbio.3002266)
Supplement: S1 Supplementary Methods — (DOCX) [file pbio.3002266.s004.docx]

# Supplementary Methods

## Chimpanzee Data

Five chimpanzees had been wild-caught and lived in captivity since then. The remaining four chimpanzees were born and raised in captivity. All subjects lived in social groups ranging from 2 to 13 individuals at Emory National Primate Research Center and were housed according to institutional guidelines. An overview of the included sample demographics is summarized in Table S1 [1].

The brains were extracted after subjects died from causes unrelated to the study. The brains were removed within 14h of each subject chimpanzee’s death and immersion fixed in 10% formalin. All brain samples were placed in agarose gel to increase mechanical stability for the MRI acquisition [1].

## Chimpanzee MRI Data Acquisition

Whole brain MRI scans of the ex-vivo samples were acquired using a 1.5 T MRI system (GE Medical Systems, Milwaukee, WI) with software version 8.3. Anatomical T1-weighted data were coronally acquired with the following parameters: TR = 666.7 ms, TE = 14.5 ms, 80 slices, slice thickness = 1.5 mm, in-plane matrix size = 256 × 256, in-plane FoV = 160 mm × 160 mm, in-plane resolution 0.625 × 0.625 mm.

**Ex-Vivo Data Challenges**

Processing ex-vivo MRI data involves specific challenges, which must be addressed to use standard in-vivo processing pipelines. First, the observed MRI contrast is affected by fixation- induced changes in relaxation times, with heavy implications on the utility of in-vivo contrast- based segmentation tools. Second, the brains are extracted and embedded in agarose with high signal intensity - making it challenging to use typically employed brain extraction tools. Third, air bubbles trapped in gyri or agarose may cause local distortions or contrast changes and need to be carefully excluded from the overall reconstruction.

## Preparing Ex-Vivo Data for FreeSurfer

We implemented a semi-automated pipeline to prepare the ex-vivo MRI of each chimpanzee to be later processed in FreeSurfer 6.0.0. In total, 5 steps were necessary: (I) Align the brains to the AC-PC convention (thus simplifying their visualization across tools), (II) separate the brain from the background using an in-house semi-supervised algorithm (to avoid errors during FreeSurfer’s brain extraction), (III) standardize the brain resolution to 0.7mm isotropic, (IV) invert the contrast on those chimpanzees with white matter darker than gray matter (FreeSurfer expects a T1-like contrast), and (V) apply a Gaussian blurring of 0.4mm to smooth intensity values within both gray-matter and white-matter tissue.

## Semi-supervised FreeSurfer Pipeline for Chimpanzees

Given that FreeSurfer was designed to process human brains, we had to use a configuration slightly different from the default to process the chimpanzee brains. Particularly, we: (1) Remove the brain extraction process, since we had already separated the brain from the background (see previous section), (2) run the whole pipeline using the “high resolution” flag, which stops FreeSurfer from resizing the voxels to 1 mm isometric, (3) removed the “Talairach check” since it fails when processing non-human brains, (4) manually normalized the brain intensity, such that the average white-matter value was 110, and (5) manually checked the white-matter segmentation, fixing it when necessary (though this is a normal step for processing humans as well). To simplify controlling the pipeline, each step was run separately and followed by a thoughtful visual control.

**Registering the Individual Chimpanzee Surfaces to a Common Template**

To compare the chimpanzees in a common space, we registered the individual brains to the JUNA chimpanzee template. Given that the JUNA template is volumetric, we first derived its 3D brain surface reconstruction with the same FreeSurfer pipeline used on the remaining chimpanzee brains. Once the reconstruction was available, we performed surface-based registration with the Multimodal Surface Matching (MSM) algorithm, using the JUNA template as the source and each individual chimpanzee brain as the target. Surface-based alignment has been proven to be more robust and precise than volumetric-based registration [2].

Since the chimpanzee brains are consistent across individuals, we used the original pairwise implementation [3], which results in a fast yet accurate registration. For the exact configuration file please see the file `chimp-to-juna-msm-config.txt` in our publicly available repository.

## Deriving the Chimpanzee Cytoarchitectural Probabilistic Atlas

By inverting the transformations derived in the previous step, we were able to project each individual cytoarchitectural segmentation to the template. Having them in a common space, we then averaged the segmentations to derive a high-quality population surface map of both BA44 and BA45 in the chimpanzee brain.

## Co-Registering the Human and Chimpanzee Brain

To enable a cross-species comparison between humans and chimpanzees, we performed an alignment between the brains of both species. Specifically, our goal was to align the JUNA chimpanzee template with the human MNI template (ICBM152 9c Asymmetric). Given the difference in volume and proportion of white/gray matter between humans and chimpanzees, volumetric registration is sub-optimal. Hence, we performed a surface-based alignment which is not subject to this restriction. Surface-based registration has been shown successful to compare brains across species, due to its ability to characterize and align brains based on their gyrification [4]. Before registering the brains, we first had to process the MNI template to reconstruct its 3D brain surface representation. This was achieved using the FreeSurfer pipeline with default parameters. FreeSurfer is perfectly suited for processing human brains, for which no inconveniences were raised during the process.

To perform the surface-based registration between the JUNA template surface and the MNI one we used the strain-based regularization version of MSM [5]. This algorithm produces smoother warps and better alignment in the presence of noise, and thus should be preferred when aligning brains across species. The registration was performed in two stages. In brief, we first aligned the brains based on gross anatomical landmarks, to then improve the alignment based on local sulcal data.

For the first stage, we derive a binary mask for the Inferior Frontal Gyrus (IFG) in both templates. The IFG is in fact extracted from the anatomical parcellation produced by FreeSurfer (known as the Desikan atlas). Since humans and chimpanzees have pronounced landmarks surrounding the IFG (Sylvian Fissure, Precentral Sulcus, and Inferior Frontal Sulcus), the Desikan atlas adequately represents it in both species [6]. We further improved this first registration by aligning the templates using local sulcal data. Since the human brain is bigger and thus has higher resolution, we performed the alignment using the MNI brain as the source and the JUNA template as the target. For the exact configuration file please see the configuration file `human- to-chimp-msm-config.txt` in our publicly available repository.

# References

1. Schenker NM, Hopkins WD, Spocter MA, Garrison AR, Stimpson CD, Erwin JM, et al. Broca’s area homologue in chimpanzees (Pan troglodytes): probabilistic mapping, asymmetry, and comparison to humans. Cereb Cortex. 2010;20: 730–742.

2. Fischl B, Rajendran N, Busa E, Augustinack J, Hinds O, Yeo BTT, et al. Cortical folding patterns and predicting cytoarchitecture. Cereb Cortex. 2008;18: 1973–1980.

3. Robinson EC, Jbabdi S, Glasser MF, Andersson J, Burgess GC, Harms MP, et al. MSM: a new flexible framework for Multimodal Surface Matching. Neuroimage. 2014;100: 414–426.

4. Eichert N, Verhagen L, Folloni D, Jbabdi S, Khrapitchev AA, Sibson NR, et al. What is special about the human arcuate fasciculus? Lateralization, projections, and expansion. Cortex. 2019;118: 107–115.

5. Robinson EC, Garcia K, Glasser MF, Chen Z, Coalson TS, Makropoulos A, et al. Multimodal surface matching with higher-order smoothness constraints. Neuroimage. 2018;167: 453–465.

6. Hopkins WD, Li X, Crow T, Roberts N. Vertex- and atlas-based comparisons in measures of cortical thickness, gyrification and white matter volume between humans and chimpanzees. Brain Struct Funct. 2017;222: 229–245.
