# Supplementary material for: Morphological evolution of language-relevant brain areas
Source: PLoS Biol. 2023 Sep 1;21(9):e3002266. doi: 10.1371/journal.pbio.3002266 (PMC10501646; doi:10.1371/journal.pbio.3002266)
Supplement: Attachment — Submitted filename: Response to Reviewers.docx [file pbio.3002266.s005.docx]

**Reply to Editor’s suggestions:**

We, the authors, would like to thank you and the reviewers for the time invested in reviewing our paper. We have now revised and improved the manuscript taking into account all the feedback provided.

Following all reviewer’s suggestions, we have now toned down the text, stating that the present data contributes to the discussion on action/language relationship, and Broca’s area evolution, rather than as a definitory solution.

We have also thoughtfully considered the comments from reviewers #2 and #3, now clearly stating the limitation of our methods, and pointing that different evolutionary scenarios are compatible with our results.

Furthermore, on revising our pipeline we found a small error when computing the average volume of the chimpanzee’s BA44 and BA45 in the human template. The error had virtually no impact in the results, but the numbers were updated for correctness.

We now provide a point-by-point answer to the all of the reviewer’s comments below.

**Reply to Reviewer #1 Comments:**

**Reviewer #1:** *This is very interesting Report, focusing on a key question in the evolution of (neurobiological bases of) language (the relationship between action and syntax), and on an area of the brain that is central to the language circuit ("Broca's region", and in particular BA44). The authors rely on histological data and cortical registration methods to offer a close comparison of the morphology of BA44 and 45 between humans and chimpanzees. Their main result is that "the left human BA44 evolved to accommodate syntax through an anterior expansion of action-related regions in the inferior frontal cortex". This is a claim that is sure to trigger a lot of discussion. The evidence provided will likely be closely examined by proponents of different scenarios for the evolution of syntax. It constitutes a genuine novel finding, in my opinion. I am therefore happy to recommend publication.*

**Reply:** We thank the reviewer for the generally positive evaluation.

**Reviewer #1:** *My only concern pertains to the rather generic use of the term "syntax" throughout the manuscript. As the authors are aware, "syntax" likely consists of multiple subcomponents/traits, and these are likely to be distributed across the brain. This, of course, does not diminish the role of BA44 is core aspects of syntax, but it may require a bit more qualification.*

**Reply:** We thank the reviewer for pointing this out. During our work we use the term “syntax” to refer the set of grammatical rules that governs how words are arranged in a phrase or sentence. In this way, syntax is a subcomponent of human language (with others being semantics and phonology), and its definition is independent of any linguistic theory.

We have now clarified this in the introduction.

**Reviewer #1:** *I also think that the results presented in this Report are unlikely to "provide a solution for the long- standing debate concerning the structural and functional evolution of Broca's area and its role in action and language". Proponents of the view that syntax evolved out of an Action Grammar capacity are likely to take these results to be evidence for a duplication-and-divergence model (evolutionary continuity as opposed to 'novelty'). I leave it to the authors to decide if their claim should be qualified. I don't feel I should insist on this modification. I think that the results are worthy of publication in PLOS Biology as they are, and whatever conceptual position the authors adopt will be adequate, as it sure will fuel discussion in the future.*

**Reply:** We thank the reviewer for pointing to the possibility of a different interpretation. We have toned down our claims, now stating that our results “add to the discussion” on the role of Broca’s area in action and language. Moreover, the discussion now clearly states that different scenarios are compatible with our results. (e.g. neural recycling, neural reuse or cultural reuse).

**Reply to Reviewer #2 Comments:**

**Reviewer #2:** *In their work, Gallardo and colleagues examine the evolution of Broca's area by comparing the cytoarchitectonic segmentations of BA44 and BA45 in humans and chimpanzees.*

*They reconstructed the cortical surface of nine chimpanzee brains using MRI data and aligned brains from chimpanzees and humans to perform a direct comparison of segmentations between the species. They found no BA45 asymmetry at chimpanzee level unlike for humans. Furthermore, they showed an expansion of Broca's areas in humans, with the left BA44 being the most enlarged. The authors conclude that BA44 in humans has evolved from a purely action-related region to a larger region, with a posterior region that supports action and an anterior region that processes syntax.*

*The results are novel and will potentially advance our understanding of the evolution of Broca's area in the primate lineage. The study is methodologically sound, and the number of animals used is sufficient for a study with non-human primates.*

**Reply:** We thank the reviewer for the generally positive evaluation.

**Reviewer #2:** *I have only one major concern regarding the overall approach that the authors should consider in their revision.*

*1. The authors strongly suggest that their data will help improve our understanding of the evolution of Broca's area in humans. Here, they argue that Broca's area may have evolved continuously from precursor structures in the primate lineage. However, to be truly convincing here, it would be essential to go back another step in evolution and look at the morphology of the monkey homolog of Broca's area. This is particularly suggested by the fact that there are a large number of studies present showing morphological and physiological similarities between the macaque monkey homolog and the human Broca's area (notably the extensive work by Pandya and Petrides, but also others). In addition, there are already several reviews addressing the evolution of Broca's area in the primate lineage. I strongly recommend that the authors consider the monkey data and incorporate and discuss the existing review articles accordingly.*

**Reply:** We thank the reviewer for the suggestion. Following the recommendation, we have now included and discussed publications concerning Broca’s area across species such as Pandya & Petrides (2009), and Zilles and Amunts (2018). In discussing the evolution of Broca’s area Zilles and Amunts (2018) argued that it is problematic to compare “the relatively distantly related macaques with humans. Instead, we must perform additional studies of cytoarchitecture and connections in our nearest relatives, that is, the bonobo and common chimpanzee…”.

Because of this, we preferred to discuss the existing reviews, without adding an explicit comparison with macaque data.

**Reply to Reviewer #3 Comments:**

**Reviewer #3:** *This short report presents a comparative analysis of human BA44/45 and their chimpanzee homologues in which they probe a hypothetical allometric expansion of these areas in humans vs chimpanzees. The principal result is that the anterior division of the area referred to as "Broca's region" in humans shows a 'disproportionately' great expansion vis-a-vis the right homologue and the more posterior subregion. The methods appear solid, the data and methods are available. The statistical analyses are acceptable. The findings are novel but their importance currently seems over-stated. The rationale for the study is that there are two opposing views on the emergence of language, either from action or independently of it.*

**Reply:** We thank the reviewer for the critical comments that we took up in a revised version.

**Reviewer #3:** *A key reference is not cited: Zilles & Amunts (2018) discuss comparisons between human and non-human primate LIFG anatomy and this work should be considered, and possibly foregrounded (especially given that it explicitly calls for a comparison between
human and chimpanzee neuroanatomy) in the introduction.*

**Reply:** We thank the reviewer for the suggestion. We now cite Zilles & Amunts (2018) and their strong plea for comparing humans to chimpanzees when considering the cytoarchitectonic structure.

**Reviewer #3:** *The authors make the bold claim that this:*

i. *Shows that BA44 "evolved from a purely action-related region"*
ii. *Solves a longstanding debate regarding the structural and functional evolution of Broca's area and its role in action and language*

*It is unclear how looking at anthropoid primates can "settle the debate". The neural basis of action and language in humans is already known (characterized to some extent, even if not completely described), it is not by examining non-human primates that these can be better elucidated.*

**Reply:** The reviewer is right in saying that the present data do not solve the debate, so we have now tone down our claims, stating that our results add to the discussion of the structural and functional evolution of Broca’s area and its role in action and language.

We further thank the reviewer for their comment, they point to the need of better explaining our motivations in the text.

While it is broadly accepted that Broca’s area supports both action and language in the human brain, it remains unclear if both domains share the exact same neural basis. Such debate arises from the (di)similarities of action and syntax. This has indeed created a discussion about the relationship between action and language. However, almost all studies have looked at either action or syntactic aspects of language separately, with only three studies directly comparing both. In this context, we do believe that examining non-human primates can be elucidating. This is because both chimpanzees and humans present a Broca’s area homolog involved in action, but only the human Broca’s area is related to syntax processing. While chimpanzees can communicate, they cannot generate or comprehend syntactic complex sentences as we humans do, therefore the extension of BA44 in humans compared to chimpanzees presented here adds to the existing data.

We have now made this statement clearer in our introduction, and reworked the discussion to clarify these points. Furthermore, we now clearly state Zilles & Amunts (2018) plead to study chimpanzee’s as a motivation for this comparison, as suggested by the reviewer

**Reviewer #3:** *The assertion that the manuscript provides "a complete picture of Broca's area evolution and … solution to the longstanding debate of BA44's role in language and action" is not well supported. The comparisons presented could not provide a full picture (but nor would they strictly need to), and moreover the function of human BA44 in language and action can only be established by studying humans. Mapping cytoarchitectonic homologues does not itself demonstrate any particular functional role and drawing inferences about the presumed functional drift of a region over evolution requires more convincing argumentation.*

**Reply:** We agree with the reviewer that the function of human BA44 can only be established by human studies. This is why we included highly relevant human studies and discuss our findings in their context. Particularly, we include and discuss the results from a meta-analysis of human functional studies (Papitto et al. 2020), and include references to studies comparing tool use (action) and syntactic processing (language) in a within-subject design (Thibault et al. 2021), and a meta-analysis (Kulik et al. 2023). All these studies support the idea that the human brain possesses an independent neuronal basis for language and action within Broca’s area. Moreover, the anterior/posterior functional division of Broca’s area that some of these papers find is supported by the receptorarchitectural division of Amunts et al. (2010) which, as stated in the paper, “is a powerful indicator of functional diversity.”

We have now added a reference to the Amunts et al. (2010) paper, and use it to backup the functional division encountered by Papitto et al.’s (2020) meta-analysis, Kulik et al.’s (2023) meta-analysis, and Thibault et al.’s (2021) action/language study. We further visually compare the receptorarchitectural maps from Amunts et al. (2010) with our results, as no volumetric data are available.

**Reviewer #3:** *If I have understood the argumentation correctly, the authors suggest that because chimpanzee BA44 maps onto only a small, posterior portion of human BA44, human BA44 has undergone an anterior expansion since the LCA with chimpanzees. This seems to depend upon an assumption that the human BA44 is a cytoarchitectonically homogeneous region. Since anterior BA44 (loosely) tends to be found to process syntax, the assumption is that the "syntax part" is derived from the "action part". This result supposedly answers the question of whether the syntax processing region in anterior BA44 evolved from action processing, and not independently. This could be a relatively compelling argument, and certainly fits with a prevailing view (as described in the introduction), but it is need of additional support.*

**Reply:** We did not make our argumentation clear enough. In our work we do not claim, expect, nor assume human BA44 to be homogeneous at all levels of analysis. In fact, the work by Amunts et al (2010) (now cited in the manuscript and visually compared with our results) shows that BA44 underlying architecture can be at least divided in two regions. Moreover, the studies of Clos et al. (2013), Papitto et al. (2020), Thibault et al. (2021), and Kulik et al. (2023) support that this division underlies a segregation of action and syntax processing.

When mapping the action-related chimpanzee BA44 to the human brain, we found it only overlaps with only maps to the “action” sub-division of human BA44, and not the “syntax” one. This further supports the idea of a bipartite system (i.e. a non-homogenous human BA44), and suggests that an anterior expansion took place to accommodate “syntax” in the human brain.

**Reviewer #3:** *On the basis of the evidence presented it would be equally possible to claim (albeit perversely) that chimpanzee BA44 evolved from a region that supported both syntactic and action processing and lost the division concerned with syntax. It seems important to consider that chimpanzees are not the human/chimpanzee LCA - and that they have presumably also been undergoing evolutionary changes over the past 5-10million years.*

**Reply:** We agree with the reviewer that our anatomical mapping cannot provide a definitive answer to the processes underwent by Broca’s area, and that multiple scenarios are compatible with our result. We have reworked our discussion to clearly state our limitations and reflect the important points raised by the reviewer.

We also now state that our work contains the implicit assumption that the chimpanzee homolog can serve as a referential model to that of our shared last common ancestor, and discuss fossil evidence that supports this assumption.

**Reviewer #3:** *Anderson (2010) proposes neural reuse as a mechanism whereby a cytoarchitectonically homologous region of human and non-human cortex could become functionally differentiated. Some kind of argumentation along these lines (or any theory the authors favor) is required to help lend credibility to the proposals.*

**Reply:** Following the reviewer’s suggestion we have now added the argument that cytoarchitectonically homologues region can be differentiated into a more posterior ventral part and a more anterior-dorsal part on the basis of receptorarchitectonic analysis (Amunts et al., 2010, PLOS Biology), thereby showing a functional separation exists. Furthermore, we now clearly state in the reworked discussion that our anatomical comparison does not allow us to distinguish the exact underlying evolutionary process that Broca’s area underwent (e.g., recycling, neural reuse or cultural reuse). Instead, we point the reader to the thorough discussion by Amunts et al. 2010 on the subject, and cite different possible scenarios from Anderson et al. 2010, Dehaene et al. 2007, and D’Ambrosio et al. 2017.

**Reviewer #3:** *I feel that there is a flaw in the current set-up of the study, but this may be a misunderstanding arising due to the density of the manuscript. Given the widely accepted assumption that chimpanzees have no language (as defined by the authors), it would be impossible for any result to arise other than one showing that chimpanzee action-related brain areas map onto human brain areas (as there are no chimpanzee language-related areas to map). It would therefore seem impossible for the authors to test the hypothesis that language arose independently of action using this method.*

*Even if Chimpanzee BA44 mapped directly onto human BA44, this would provide evidence for or against - it would merely indicate that some functional modification of a previously homologous region must have taken place. In the present case it is hypothesized that there was an expansion, but if that expansion could only be found to have arisen from a region that is characterized in chimpanzees and therefore non-linguistic, it does not seem that the debate has been solved. If this is not the case, it would be very important to discuss which result would lead to the opposite conclusion. Mapping cytoarchitectonic probability maps from one atlas to another using the methods employed here could never lead to the discovery of an area in one species that does not map onto some region of the other.*

*Thus, the results regarding the extent of allometric expansion and the asymmetry are themselves compelling, but the interpretation is pushed too far.*

**Reply:** We agree with the reviewer that the density of the manuscript could lead to misunderstandings. We have modified the text to clarify that we are not stating (nor testing) that language “arose independently” from action. Our aim instead is to use cross-species comparison to support the claim that action and syntactic-processing are represented independently in human Broca’s area.

An important remark is that Chimpanzees do possess some aspects of language (e.g. they can communicate using gestures). However, they do not generate nor comprehend utterances in the form of complex syntactical structures as humans can do. Since chimpanzees possess a Broca’s area homolog related to action and gestures, and Broca’s region in humans is related to action and syntax, comparing both species can shed light into the spatial differences, and thus, the changes underwent by Broca’s region (once more, assuming a close relationship between chimpanzee’s Broca’s and our last common ancestor).

By using a state-of-the-art method of anatomical mapping based on scaling and displacement we aligned the chimpanzee and human brain. This allowed us to directly compare Broca’s area across the species. What we found is that the chimpanzee’s Broca’s area maps to the “action-related” section of human’s Broca’s area, and not syntax. This means that a simple anatomical scale and shift of the chimpanzee’s Broca does not explain the existence of the anterior syntax sub-region of Broca’s area in the human brain. We further compared the lateralization and relative-size of Broca’s area across species to confirm that its cythoarchitectural sub-divisions (i.e., BA44 & BA45) not only expanded, but did it in a disproportional way, with BA44 becoming left lateralized and expanding anteriorly.

We further agree with the reviewer that our original discussion pushed the result too far. Now we have toned down our claiming, and say that, in conjunction with functional studies, our results present evidence in favor of indicating a current independence between syntactic aspects of language and action, while also clearly indicating our study’s limitations.

**Reviewer #3:** *To sum up - I am not opposed to the authors' position that language conceivably arises on the basis of action systems. However, I do not believe that the present paper demonstrates this as conclusively as claimed. The pattern of allometric expansion from chimpanzee to human with a greater expansion in the left than right hemisphere and a greater expansion in BA44 than 45 is extremely intriguing and these analyses are of interest to the community of researchers concerned with the evolution of the neurobiological bases of language. However, the argumentation is under-developed, limitations and fundamental assumptions relevant to the approach are not really discussed and there is no convincing control analysis that can help to support that the pattern of results is related to language specifically. My recommendation is therefore for substantial modification to the introduction and discussion to justify the approach and the strength of the claims.*

**Reply:** We thank the reviewer for the thoughtful and useful feedback. We have taken into account all the suggestions raised, revising our argumentation and claims in the text accordingly.
