# Supplementary material for: Morphological evolution of language-relevant brain areas
Source: PLoS Biol. 2023 Sep 1;21(9):e3002266. doi: 10.1371/journal.pbio.3002266 (PMC10501646; doi:10.1371/journal.pbio.3002266)
Supplement: Attachment — Submitted filename: Response to Reviewers.docx [file pbio.3002266.s006.docx]

**Response Letter**

We, the authors, would like to thank all reviewers once again for the time invested in reviewing our paper. We are happy that reviewers #1 and #3 are satisfied with the revised version, and we understand reviewer #2 request for discussing monkey data.

We have thoughtfully considered each of the papers listed by reviewer #2, which discuss the possible precursors of speech, and added a new paragraph discussing our results in light of functional and structural brain data in monkeys.

We thank reviewer #2 for the suggestion to include a discuss monkey data to strength our paper.

**Paragraph added in Discussion:**

*Along with contributing to the debate on the relationship between action and language, our findings add to the broader topic of language origins. Although there is an evolutionary continuity in auditory-vocal processing and their underlying neurobiological substrate in temporo-frontal networks [39–42], functional neuroanatomical changes appear to play a crucial role during the evolution of prefrontal cortex. Functional studies indicate that when macaques process simple grammatical sequences activation of both Broca’s area and the frontal operculum is observed [43]. In contrast, human brains solely recruit the frontal operculum for simple grammars, whereas BA44 comes into play when processing complex grammatical sequences that non-human primates cannot process [44,45]. Thus, despite the observed similarity in the organizational principles across primates, it may have been the expansion of cytoarchitectonically-defined BA44 throughout evolution that paved the way for the representation of language in the human brain.*

**References added**:

*39. Petkov CI, Jarvis ED. Birds, primates, and spoken language origins: behavioral phenotypes and neurobiological substrates. Front Evol Neurosci. 2012;4: 12.*

*40. Aboitiz F. Voice, gesture and working memory in the emergence of speech. Interact Stud. 2018;19: 70–85.*

*41. Rauschecker JP. Where did language come from? Precursor mechanisms in nonhuman primates. Curr Opin Behav Sci. 2018;21: 195–204.*

*42. Hage SR, Nieder A. Dual neural network model for the evolution of speech and language. Trends Neurosci. 2016;39: 813–829.*

*43. Wilson B, Kikuchi Y, Sun L, Hunter D, Dick F, Smith K, et al. Auditory sequence processing reveals evolutionarily conserved regions of frontal cortex in macaques and humans. Nat Commun. 2015;6: 8901.*

*44. Friederici AD, Bahlmann J, Heim S, Schubotz RI, Anwander A. The brain differentiates human and non-human grammars: functional localization and structural connectivity. Proc Natl Acad Sci U S A. 2006;103: 2458–2463.*

*45. Fitch WT, Hauser MD. Computational constraints on syntactic processing in a nonhuman primate. Science. 2004;303: 377–380.*
